# Supplementary material for: Effectiveness and Safety of Ayurvedic Medicines in Type 2 Diabetes Mellitus Management: A Systematic Review and Meta-Analysis
Source: Front Pharmacol. 2022 Jun 8;13:821810. doi: 10.3389/fphar.2022.821810 (PMC9213670; doi:10.3389/fphar.2022.821810)
Supplement: Supplementary file 1 [file DataSheet1.zip › Supplementary Material/Forest and Funnel Plots/Sesamum indicum/Sesamum indicum.pdf]

Outcomes	Number of studies	Mean Diff. with 95% CI	Heterogeneity (%)
HbA1c	2	-0.70 [-1.42, 0.03]	23.46
FBG	2	-45.50 [-115.80, 24.80]	95.26
Fasting insulin*	2	8.30 [-6.97, 23.58]	86.66
Insulin resistance	1	0.54 [-3.42, 4.50]	N/A
Body weight	1	0.79 [-7.77, 9.35]	N/A
BMI	1	0.96 [-2.21, 4.13]	N/A
Waist circumference	1	3.06 [-3.68, 9.80]	N/A

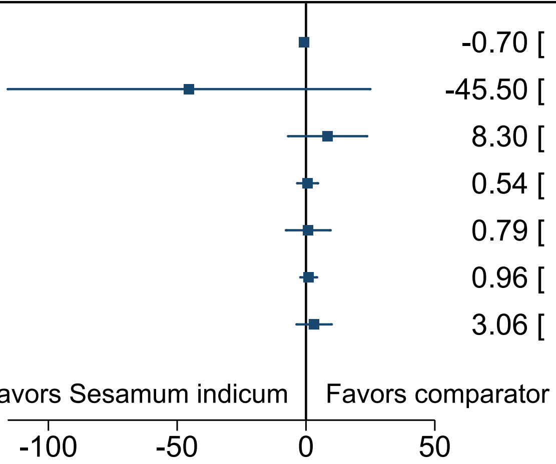

*Favors intervention/comparator as insulin sensitizer
